# A model for faculty engagement in distributed medical education: Crafting a paddle

**Published:** 2018-03-27

**Authors:** Barbara Zelek, James Goertzen

**Affiliations:** 1 Section of Family Medicine, Northern Ontario School of Medicine, Ontario, Canada

## Abstract

Distributed medical education initiatives are now a key component of all Canadian medical schools. The success of these initiatives requires engaged community-based faculty who are able to successfully balance both their clinical and educational responsibilities. Present understanding of faculty engagement within distributed medical education is limited. Faculty engagement is a complex and multifaceted construct that includes a reciprocal relationship between a Faculty of Medicine and their faculty. Clarification of both the extrinsic and intrinsic motivators of distributed faculty provide opportunities for Faculties of Medicine to more fully engage their faculty and sustain distributed medical education programs.

## Introduction

I was a busy rural community preceptor when all of a sudden the tsunami of the distributed medical campus hit. Now I’m wet and I’m cold. In addition to providing care to my patients, I’m expected to teach these students and to swim in competency based education. All I have is my PFD (Personal Flotation Device) covered in PDFs (Portable Document Formats) of curriculum expectations, field notes, and evaluations. Am I sinking or swimming? My paddle is broken and I wonder how the medical school will help me build a new one so that I can balance both my clinical and teaching responsibilities.

The Association of Faculties of Medicine of Canada Distributed Medical Education Resource Group (AFMC DME) identified faculty engagement in DME as a priority recognizing that engaged faculty are crucial to the development and delivery of ongoing distributed medical education.^1^ In 2014, an Engagement Working Group was tasked to review the literature on faculty engagement, develop a definition, clarify metrics to measure engagement, and identify strategies to support faculty engagement.

This AFMC DME Working Group recognized that for distributed medical education there is no clear definition of faculty engagement.

Nevertheless, to address this gap in our understanding about faculty engagement in distributed medical education we drew on some of the salient literature in business, health care and higher education to summarize the state of faculty engagement in medical education. Using Pink’s^2^ tenets of motivation: autonomy, mastery, and purpose, we will provide a framework for Faculties of Medicine to engage their distributed faculty. We propose a definition of faculty engagement in distributed medical education and using this framework we will provide our fictional exasperated physician and their medical school with plans to build a new paddle and together travel in the turbulent waters of clinical care and medical education.

## Distributed medical education

To address rural physician shortages, expand physician training capacity and embrace social accountability, Canadian medical schools are increasingly relying on distributed and community-based settings as key educational sites^3^. Distributed training models have resulted in a proliferation of distributed training sites, burgeoning regional medical campuses in all faculties of medicine, and the establishment of the Northern Ontario School of Medicine (NOSM), whose campus is all of Northern Ontario.^3,4^

Distributed approaches to medical education have required a considerable increase in part-time distributed clinical faculty affiliated with Canadian medical schools. In Canada, the number of part-time faculty members increased dramatically between 2003 and 2007 and 2015, from 16,061 to 21,687 to 37,984. During this time period, part-time family medicine faculty members increased from 3,605 to 5,901 to 11,268. Other general speciality disciplines including paediatrics, emergency medicine, internal medicine and general surgery have also seen similar increases in part-time faculty.^4,5^

Distributed training models that include both undergraduate and postgraduate educational activities have the greatest potential to graduate physicians with the skills, knowledge and attitudes to practice in rural and remote settings.^6^ The development and sustainability of these educationally sophisticated training sites require faculty who, in addition to the provision of clinical care to their communities also engage in educational activities. These include precepting a wide range of medical and health professional learners, assisting with both development and implementation of educational curricula, and generally supporting the educational mandate of their faculty of medicine.

## The problem defined

Most distributed faculty see themselves as clinicians first with a secondary role as teachers.^7^ They have a commitment to supervising medical learners as they provide quality clinical care to their community. In discussions with distributed faculty, often their commitment to teaching is driven by a goal of ultimately improving local clinical human resource needs. Little is known about how these faculty members define themselves within their academic or faculty roles or how they engage with their affiliated medical school. In addition, there is limited understanding and research on how medical schools define, measure, or increase the engagement of their distributed faculty.

## Defining engagement

The literature on employee engagement may provide the foundation for understanding engagement in the medical education context. Employee engagement is identified as being a multifactorial process influenced by both enablers and disenablers.^8^ In addition, employee engagement includes emotional, rational, and behavioral components.^9^ Engaged employees are more likely to actively further the organization’s interests through their enthusiasm and interest in their work. Spurgeon et al.^10^ define physician engagement in the clinical or hospital context as “the active and positive contribution of doctors within their normal working roles to maintain and enhance the performance of the organization itself.” Building upon this definition, Clark^11^ contends that physicians are engaged if they consistently say positive things about their organization as a place to work; if they intend to stay and continue to practice at the organization; and if they strive to achieve beyond their expected daily role. The “organization” can be defined as either the hospital as a “workplace” when referring to physician engagement in a clinical sense, or could be extrapolated to be an academic institution in medical education and their relationship with medical education faculty. Due to the part-time nature of distributed faculty and their more tenuous relationship with their academic institution, it seems likely that more effort is required from the institution over time to ensure ongoing alignment and engagement.

Physician engagement in hospitals is important because it is correlated with higher performance and innovation, higher scores on a variety of quality indicators including decreased patient mortality and decreased incidents of patient harm, as well as improved hospital financial performance^12^ and improved patient satisfaction.^13^ Furthermore, it is tied to other concepts crucial to performance outcomes: physician satisfaction, commitment, and leadership.^14^ One can surmise this literature is relevant to distributed faculty working in hospital settings and affiliated as faculty members with their medical school.

Literature describing engagement concepts in higher education is complex due to a range of behavioral, psychological, socio-cultural, and holistic perspectives.^15^ The concept of engagement for the teacher or learner with their educational institution is a dynamic continuum that includes affective, cognitive, and behavioral factors. For physicians, engagement within their medical school is a developmental process as their professional identity incorporates their roles as teachers. This is important as physicians who see themselves as teachers are more likely to enjoy teaching, teach more and be recognized by students as good teachers.^16^ Furthermore, when faculty identify themselves as teachers, this identification may influence their desire to teach and improve their teaching skills, satisfaction with teaching and, ultimately, student learning.^17^

In summary, business, health care, and higher education literature findings suggest that faculty engagement is a complex and dynamic relationship with both enablers and dis-enablers. Increased faculty engagement is likely associated with higher performance and satisfaction by individual faculty members. More theory development is required and we propose using motivation theory as a framework.

## Motivation theory as a framework for faculty engagement in DME

Pink^2^ has reviewed the current research on human motivation and posits that intrinsic human motivators (purpose, mastery, and autonomy) are more important and, in fact, more inherent motivating forces than extrinsic motivators. Extrinsic motivators, such as salary, recognition, and reward are important but clearly not the only or the strongest motivators. We will make the argument that human motivation theory as presented by Pink can be used as a framework to understand faculty engagement in DME and the situation surrounding our struggling faculty member.

Zelek and Rossi conducted a facilitated discussion of NOSM faculty exploring faculty engagement amongst distributed physician faculty.^18^ This nominal group identified factors that affect faculty engagement including those that can be categorized as extrinsic motivating factors such as stipends, recruitment of new colleagues, recognition by the school, access to continuing medical education, library access, and promotion. The group also identified factors that would be considered as intrinsic: the enjoyment of teaching, giving back to the profession, helping to shape the next generation of medical experts, and their personal identification as teachers (e-mail Workshop Summary to participants, March 31, 2015; unreferenced).

Further support of the relevance of internal motivators for physician engagement comes from Grimes and Swettenham^19^ who identify drivers of physician engagement that include, among other things, having confidence in the school’s success, believing that the school cares about its faculty, feeling students are satisfied with the quality of teaching they receive, and believing that the organization treats faculty with respect. Clearly physicians do what they do for reasons bigger than themselves and their financial rewards.

Both the NOSM faculty group^18^ and the research by Grimes and Swettenham^19^ support Pink’s^2^ tenet of internal motivation as a driving force for engagement. The specific intrinsic motivators identified in both the literature and the NOSM group can be further categorized into Pink’s nomenclature of purpose, mastery and autonomy.

### Purpose

The intrinsic motivator of PURPOSE is the dominant theme that arises in both our search of the literature^9^ and in our own nominal group.^18^ Pink^2^ argues that the most deeply motivated people hitch their desires to a cause larger than themselves, emphasizing meaning, significance, and contributions to the world. Individuals with a sense of purpose report higher levels of satisfaction and subjective well-being and low levels of anxiety. By nature as humans we seek this sense of purpose. We suggest to faculties of medicine that to successfully engage faculty they must primarily tap into this sense of PURPOSE in their distributed faculty.

### Mastery

Pink^2^ defines MASTERY as the urge to get better or develop skills at something that matters. According to Pink, engagement can produce mastery. Faculties of medicine have the opportunity to provide relevant MASTERY for their distributed faculty that would include clear expectations around teaching assignments, intentional feedback to faculty for teaching improvement, access to relevant, high quality faculty development, and intentional mentorship. Significant barriers to faculty engagement were also identified by NOSM distributed faculty including lack of feedback about teaching, lack of recognition of teaching effort, lack of access to meaningful faculty development and clinical teaching resources, as well as unclear organizational expectations of them as faculty.^18^ Back to our earlier metaphor, distributed educators have the desire to tackle the waters of teaching, but they feel adrift without the proper tools; in essence, our distributed educators feel they require assistance in MASTERY.

### Autonomy

Pink^2^ argues that our default setting is to be autonomous and self-directed. In addition, in response to examining the challenges and opportunities arising as physicians engage in health system transformation, the Canadian Medical Association has recently examined the state of the professional relationship between physicians and the health care system. They propose the “AAA” (Autonomy, Advocacy, Accountability) lens.^20^ The first of these is AUTONOMY, reinforcing the value physicians place on their personal and professional autonomy. It is essential that distributed faculty maintain their sense of autonomy while delivering the academic and scholarly work of the Faculty of Medicine. Promoting autonomy for distributed faculty in their various roles includes respectful entrustment by the Faculty of Medicine that distributed faculty have specific expertise for delivery of educational activities within their setting. This type of relationship nurtures purpose, mastery and autonomy for distributed faculty.

## Next steps

AFMC DME Working Group members are conducting a qualitative study with DME program directors and faculty to further identify factors that impact faculty engagement in Canadian medical schools. Early thematic analysis identified a number of extrinsic and intrinsic motivators that act as either barriers or facilitators of faculty engagement. Within both the clinical and education setting, administrative support, recognition, and feedback from the medical school are key. Faculty are influenced both positively and negatively through their relationships with colleagues and learners along with medical school administrators and support staff.^18^ Consistent with Pink’s^2^ framework, this work reinforces that DME faculty see their educational activities most influenced by intrinsic motivators rather than extrinsic factors.

## Defining faculty engagement in DME

Bringing together the literature on engagement and motivation theory, we propose the following definition of faculty engagement in distributed medical education:

Faculty engagement is the reciprocal relationship that exists between a Faculty of Medicine that actively listens and responds to their distributed faculty and the distributed faculty who view their academic/scholarly activity as an integral part of their professional lives.

### Conclusion

Physician engagement is a complex and multifaceted construct. Canadian medical schools’ commitment to social accountability and ongoing changes to health care delivery further reinforce the important role of faculty engagement and leadership in the delivery of relevant physician training. We propose that by better understanding both the extrinsic and more importantly crucial intrinsic motivators of distributed faculty, faculties of medicine can intentionally foster a sense of purpose with their distributed faculty, who maintain their autonomy and provide the opportunity for mastery that will build faculty engagement as sustainable stewardship of our future medical education enterprise. Physician educators in distributed settings are likely to find themselves challenged by the demanding clinical and educational workloads, however, together with a responsive medical school, they can craft suitable paddles to effectively traverse the waters of clinical and faculty responsibilities.
